# Adenosine Deaminase as a Biomarker of Tenofovir Mediated Inflammation in Naïve HIV Patients

**DOI:** 10.3390/ijms21103590

**Published:** 2020-05-19

**Authors:** Francisco Miguel Conesa-Buendía, Patricia Llamas-Granda, Patricia Atencio, Alfonso Cabello, Miguel Górgolas, Raquel Largo, Gabriel Herrero-Beaumont, Aránzazu Mediero

**Affiliations:** 1 Bone and Joint Research Unit, IIS-Fundación Jiménez Díaz University Hospital-UAM, 28040 Madrid, Spain; fmconesa91@gmail.com (F.M.C.-B.); patricia.llamas@quironsalud.es (P.L.-G.); rlargo@fjd.es (R.L.); gherrero@fjd.es (G.H.-B.); 2 Internal Medicine, Infectious Diseases Division, Fundación Jiménez Díaz University Hospital-UAM, 28040 Madrid, Spain; patricia.atencio@quironsalud.es (P.A.); acabello@fjd.es (A.C.); mgorgolas@fjd.es (M.G.)

**Keywords:** tenofovir, adenosine deaminase, HIV, biomarker, inflammation

## Abstract

Plasma levels of adenosine deaminase (ADA), an enzyme that deaminates adenosine to inosine, are increased during inflammation. An increase in ADA activity occurs with lower human immunodeficiency virus (HIV) viral load and higher CD4^+^ T cell counts. We aimed to investigate the role of plasma ADA as a biomarker of inflammation in treatment-naïve HIV patients who received tenofovir or another nucleoside analog for comparison. Ninety-two treatment-naïve patients were included in the study and grouped by treatment, i.e., tenofovir disoproxil fumarate (TDF), tenofovir alafenamide (TAF) or Triumeq. ADA activity was measured in plasma and cytokines were analyzed by MILLIPLEX^®^ MAP-Luminex^®^ Technology. Plasma concentration of monocytes and neutrophils was measured at 0, 3, and 12 months post-treatment. Treatment-naïve HIV patients had increased ADA concentrations (over 15 U/L) that decreased after treatment with TAF and Triumeq, though this did not occur in TDF-treated patients. However, all groups exhibited a pro-inflammatory systemic profile at 12 months of treatment. Plasma GM-CSF levels decreased after 12 months of treatment in the TDF group, with a concomitant decrease in blood monocyte count, and a negative correlation with ADA values was found. In conclusion, ADA levels may be modulated by antiretroviral therapy in HIV patients, possibly affecting inflammatory status.

## 1. Introduction

Complex interactions between immune cells and soluble factors are responsible for the inflammatory response triggered to protect against microorganisms or at the site of injury. One such soluble factor is adenosine, a purine nucleoside that, when accumulated in the extracellular space, modulates the immune response and prevents inflammatory tissue damage [1]. The immunomodulatory capacity of adenosine has been known since the 1970s, when its role in the development and activity of several immune cells was first established. Inflammation produces an increase in extracellular adenosine, with levels reaching micromolar range [2]. These adenosine levels are regulated by a variety of mechanisms, including nucleoside transporters, intracellular and extracellular biosynthesis, and conversion to inosine by adenosine deaminase (ADA) [1]. Impaired ADA activity directly correlates with defective adenosine metabolism as shown in ADA-SCID, a severe, congenital combined immunodeficiency [3].

ADA regulates extracellular adenosine levels and consequently controls adenosine receptor stimulation and balances the immunosuppressing effects of adenosine, thus making it an indicator of cellular immunity. ADA is essential for proliferation and differentiation of lymphoid cells, especially T cells, and aids in the maturation of monocytes into macrophages [4]. Adenosine deamination is performed by ADA1 isoenzyme in T cells and natural killer cells (NK), and ADA2 isoenzymes in monocytes, dendritic cells (DCs), B cells, neutrophils, and CD26-Tregs [5]. Lymphocytes and monocytes have the highest ADA activity, up to 10 times higher than any other kind of cell [6]. ADA modulates purinergic responses to several pathophysiological events, such as rheumatoid arthritis (RA), chronic pulmonary diseases, sepsis, and inflammatory bowel diseases [7]. ADA levels are increased in rheumatoid arthritis, psoriasis, sarcoidosis, some cancers, and tuberculosis [7,8]. During inflammation, plasma levels of ADA are increased in response to higher levels of adenosine. Serum levels of ADA are assessed as an inflammatory marker and mirror monocyte/macrophage activation during inflammatory diseases [9]. Elevated ADA activity is present in the serum and synovial fluid of RA patients, with a concomitant reduction in adenosine levels owing to increased ADA activity, indicating a link between the activity of this enzyme and RA-related inflammation [10].

The main cellular targets of HIV (human immunodeficiency virus) are CD4+ cells, especially the CD4 subset of T cells, peripheral blood monocytes, and tissue resident macrophages [11]. Defective immunological function of both T cells and macrophages together with the reduction in the number of CD4+ T lymphocytes are responsible for HIV-associated immune deficiency [12]. The ability of HIV-1 to infect cells depends on T-lymphocyte activation and monocyte/macrophage differentiation stage [13]. This indicates that cytokines and growth factors can modulate HIV-1 infection and replication. Pro-inflammatory cytokines, such as interleukin IL1, IL6, and tumor necrosis factor-alpha (TNFα) can induce transcription of latent HIV-1 [14,15]. It has been described that during HIV-1 infection, type 2 cytokines (IL4, IL6, and IL10) predominate over type 1 cytokines (such as IL12, INFγ, and IL2) [16,17] although this is not widely recognized [18]. Chronic immune hyperactivation and raised T-cell turnover due to continued viral replication and antigenic stimulation are present even after HAART (highly active antiretroviral therapy) has decreased the viral load to undetectable levels [19], suggesting that unresolved inflammation during viral latency can promote the replenishment of the HIV reservoir in tissues [20]. TDF is a nucleotide reverse transcriptase inhibitor (NRTI) that, in addition to displaying anti-HIV-1 activity, is able to alter cytokine expression. In murine macrophages, tenofovir induces *IL1β*, *TNFα*, *MIP1α/CCL3*, and *IL10* mRNA expression [21] and expression of MIP1α/CCL3 and RANTES/CCL5 has been found in human peripheral blood mononuclear cells [22].

In HIV, the viral glycoprotein gp120 prevents interaction between ADA and the costimulatory signal for TCR-mediated T cell activation (CD26) [23,24]. Lower HIV viral load and higher CD4^+^ T cell counts are strongly correlated with an increase in ADA [25].

HIV patients have a higher frequency of Treg cells expressing CD39 (an ectonucleotidase that converts ATP and ADP into AMP). In these patients, effector T cells show higher in vitro sensitivity to the suppressive effect of adenosine due to higher expression of the adenosine A2A receptor [26]. Moreover, different *CD39* gene polymorphism modify the progression to AIDS, thus implicating adenosine production in disease progression [26]. Ex vivo ADA is able to enhance HIV-1 effector responses [27].

In the present study, we determine whether plasma levels of ADA can be used as a marker of the disease. Furthermore, we study cytokine levels and their relationship to inflammatory response after 12 months of treatment with tenofovir compositions TDF and TAF compared to another nucleoside antiretroviral drug (abacavir, one component of Triumeq) in naïve HIV patients.

## 2. Results

### 2.1. Comparative Evolution of ADA Expression at 3 and 12 Months after Treatment Initiation

**Figure 1:**
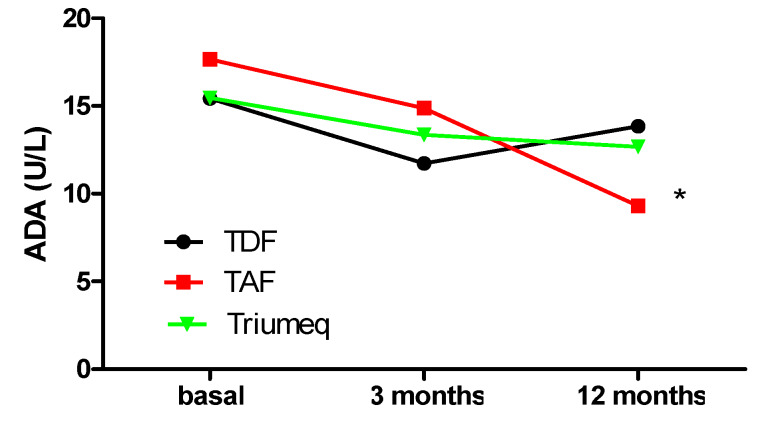
Evolution of adenosine deaminase (ADA) expression at 3 and 12 months after treatment initiation. The graph shows median ADA values in U/L for TDF, TAF and Triumeq. * *p* < 0.05 vs. baseline

In order to understand the behavior of ADA levels in treatment-naïve HIV patients and determine whether treatment with tenofovir modified these values, ADA concentration was evaluated at baseline and then at 3 and 12 months after treatment. Figure 1 and Table 1 show elevated ADA concentrations before the initiation of treatment, over normal human clinical reference limit of 15U/L. In all cases, these levels dropped 3 months after treatment was begun; this decrease was not statistically significant vs. baseline levels. When ADA was analyzed 12 months after treatment initiation, a marked decrease was observed in TAF-treated patients (9.30 (7.95, 12.72) U/L); these values reflect median and interquartile ranges (maximum and minimum). None of the patients in this group were over the limit of 15 U/L when compared to baseline levels (17.66 (13.50, 22.31) U/L) (*p* < 0.05) (Figure 1, Table 1). On the contrary, TDF produced an upward trend in ADA values from 3 to 12 months of treatment (*p* = ns), at which time 50% of patients were over the limit of 15 U/L (13.84 (12.53, 16.69) U/L) (*p* = ns. vs baseline); Triumeq remained stable (12.68 (11.59, 14.75) U/L) (*p* = ns vs. baseline), with only 20% of patients over the limit (Figure 1, Table 1). When we compare the evolution in ADA activity between treatments (increase (positive number) or decrease (negative number) compared to baseline level), we observed statistically significant changes among groups. TAF and Triumeq caused a decrease in ADA values both at 3 and 12 months that was statistically significant at 12 months (*p* < 0.0001) (Supplementary Table S1). TDF caused a statistically significant decrease when compared to TAF and Triumeq (Supplementary Table S1), although ADA evolution in TDF treated patients did not change when compared to baseline levels.

### 2.2. Comparative Evolution of Cytokine Expression at 12 Months after Treatment Initiation

Twelve months after antiretroviral treatment initiation, we observed a predominant pro-inflammatory profile among patients treated with either form of tenofovir (TDF and TAF) and, to a lesser extent, with Triumeq.

When we analyzed each cytokine individually, we observed a clear upward trend in the three treatment groups for IL2, IL6, and IL8 with respect to baseline levels, although this increase was not statistically significant (Table 2). Interestingly, other pro-inflammatory cytokines such as TNFα, INFγ, and IL1β showed variation between treatments: INFγ showed a tendency to increase by 19.2 and 30.5 times in patients treated with TDF and TAF, respectively (*p* = ns); meanwhile, INFγ decreased 11-fold in patients treated with Triumeq (*p* = ns) when compared to baseline levels (Table 2). Moreover, patients treated with TAF had the highest expression of proinflammatory cytokines after 12 months of treatment (with the exception of IL12), although changes between groups were not significant (Table 2), with the exception of IL8 (*p* = 0.015 TAF vs Truimeq; Table 2). Interestingly, IL12 decreased at 12 months in all three treatments and proportionally according to the drug used: 4-, 15-, and 36-fold for TDF, TAF, and Triumeq, respectively (Table 2).

Regarding the average concentration of anti-inflammatory cytokines in plasma, both IL4 and IL10 showed decreased expression with the three treatments studied (Table 2). Interestingly, the change in IL10 was significant when we compared TAF vs. Triumeq (7 times less than baseline for TAF vs. 44.1 times less than baseline for Triumeq, *p* = 0.036) (Table 2). No significant changes were observed in anti-inflammatory cytokines between TDF and TAF. IL13 presented a very variable expression profile between treatments: it increased 13.6 times with TDF and 32.6 times in the case of TAF, decreasing 43.5-fold when treated with Triumeq (Table 2). Comparing the change of expression for IL13 between treatments, we observed that this difference was significant between TAF and Triumeq (*p* = 0.035, Table 2).

We measured three cytokines involved in the stimulation of granulocytic colonies, that is, IL5, IL7, and GM-CSF. GM-CSF had decreased significantly at 12 months of treatment in TDF-treated patients when compared to baseline (−35.1 times, *p* < 0.0001), together with a tendency to decrease in IL5 (-17.12 times, *p* = ns). On the other hand, IL7 increased 56.2-fold when compared to baseline (*p* > 0.05) (Table 2). This decrease in colony stimulating factors was equally noticeable in the group of patients treated with Triumeq for the three cytokines studied, although these changes were not significant when compare to baseline. Changes in IL5 were almost significant (*p* = 0.055) when compared to TAF (Table 2). Surprisingly, patients treated with TAF behaved differently, as 12 months after treatment initiation, IL5 was increased when compared with baseline (78.4 times, *p* < 0.05) and GM-CSF showed a tendency to increase (7.5 times, *p* = ns). For its part, IL7 expression decreased 26.4 from baseline (*p* = ns) (Table 2). These changes in IL7 and GM-CSF were significant when TAF group was compared with TDF group (*p* = 0.01 and *p* = 0.009, respectively; Table 2).

**Figure 2:**
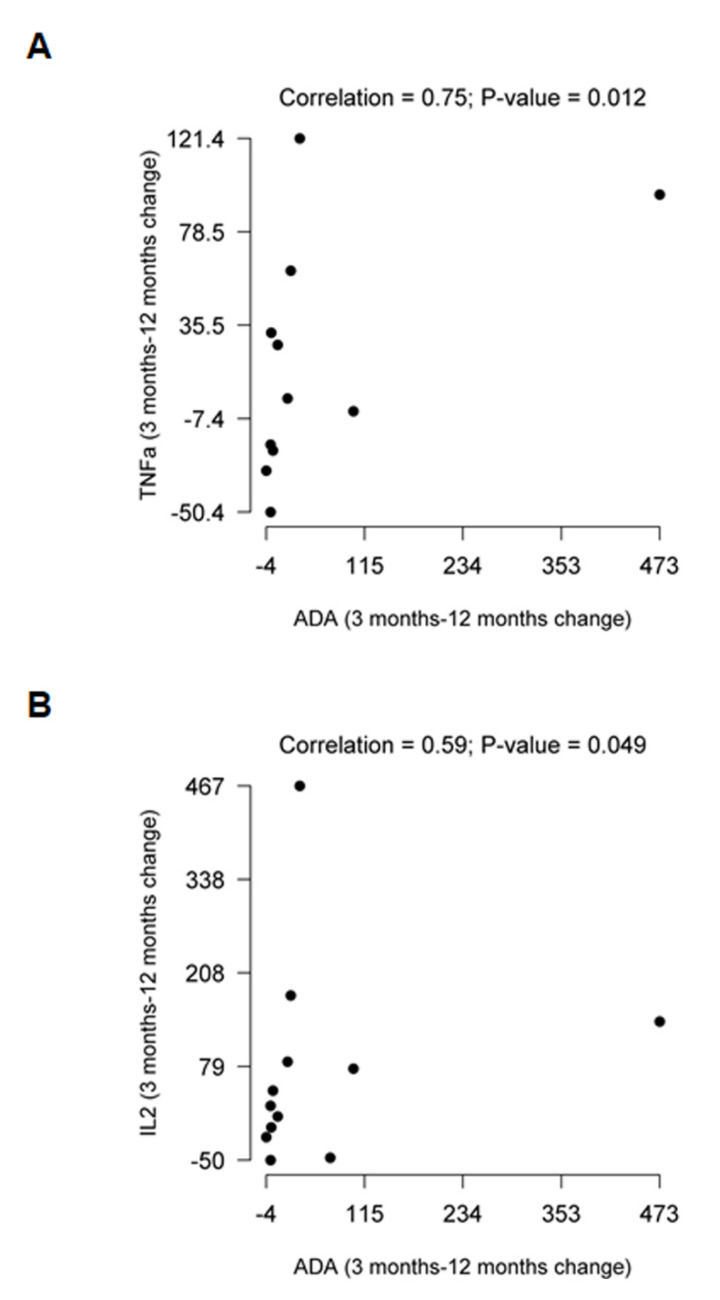
Association between ADA, TNFα, and IL2. (**A**) Spearman correlation between ADA and TNFα changes at 3- and 12-months post-treatment. (**B**) Spearman correlation between ADA and IL2 changes at 3- and 12-months post-treatment.

When we determined whether there was an association between ADA and the cytokines studied, Spearman correlation coefficient found a statistically significant positive association only between ADA values and TNFα and ADA and IL2 in TDF-treated patients when 12 and 3 months post-treatment were compared (*ρ* = 0.75 *p* = 0.012 and *ρ* = 0.59 *p* = 0.049) (Figure 2).

### 2.3. Changes in Monocytes and Neutrophils Within the 12 Months of Treatment

**Figure 3:**
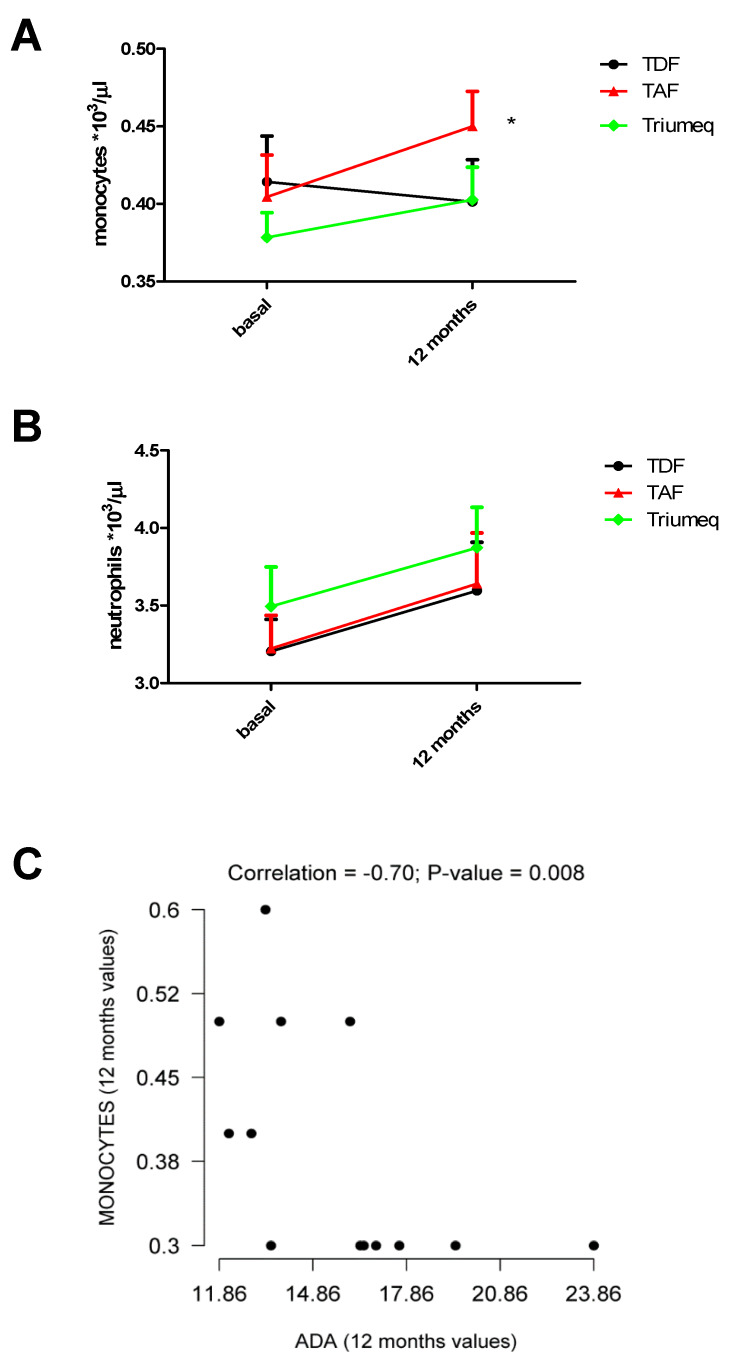
Monocyte and neutrophil counts at baseline and 12 months after antiretroviral treatment. (**A**) Changes in the number of monocytes for TDF, TAF, and Triumeq. (**B**) Changes in the number of neutrophils for TDF, TAF, and Triumeq. (**C**) Spearman correlation between ADA values and number of monocytes 12 months after treatment initiation * *p* < 0.05, *t*-student.

As ADA is essential for the proliferation and maturation of monocytes to macrophages and given the fact that we found the tendency to decrease in colony stimulating factors, specifically IL5 and GM-CSF, we decided to evaluate possible changes in monocytes and neutrophils over time. As can be observed in Table 3 and also in Figure 3A, we observed an increase in monocytes in the TAF-treated group and a tendency to increase in the Triumeq-treated group when compared to baseline (TAF: 450 cell/µL vs. 404 cell/µL at baseline level, and Triumeq: 403 cell/µL vs. 378 cell/µL at baseline levels, *p* < 0.05 and *p* = ns, respectively). Interestingly, in the case of TDF, the number of cells remained fairly stable (401 cells/µL vs. 414 cell/µL at baseline level, *p* = ns) (Table 3, Figure 3A).

When we evaluated neutrophil concentration in plasma (Table 3 and Figure 3B), we observed that the number of cells was very homogeneous in all treatment groups, increasing in a similar pattern 12 months after treatment initiation (Table 3 and Figure 3B). No significant changes between treatments were observed.

We observed a negative association between monocyte number and ADA values in TDF-treated patients at 12 months of treatment (*ρ* = –0.70 *p* = 0.008) (Figure 3C).

## 3. Discussion

In this prospective study, we studied, on the one hand, cytokine levels as inflammatory response variables after 12 months of treatment with tenofovir compositions (TDF and TAF) in previously naïve HIV patients compared to another nucleoside antiretroviral drug (Triumeq), and on the other, we studied plasma levels of ADA to investigate whether this value can be used as a marker of the disease. We observed an increase in ADA values (above normal) during the 12 months of treatment in the presence of TDF that did not occur with TAF or Triumeq. We also found a proinflammatory profile in TDF- and TAF-treated HIV patients, with a decrease in GM-CSF for TDF group.

Unlike previous studies, our work reports differences in ADA values and cytokine expression in naïve HIV-infected individuals, focusing on early treatment time points (3 and 12 months). Previous research has assessed circulating inflammatory mediators based on their reported associations with systemic inflammation [28].

In physiologic conditions, adenosine levels in the extracellular space are low, and they increase under tissue damage, inflammation, infection, hypoxia, and stressors [28]. Adenosine levels regulate function, proliferation, and activation of immune cells, and regulation of adenosine levels and its receptor activation is an efficient mechanism for the limitation and resolution of inflammation [28]. One of the regulatory mechanisms of adenosine concentration acts by converting adenosine into inosine by ADA.

In HIV, purine metabolism is involved in the onset of infection, the maintenance of inflammation, and in immune activation. HIV infection induces changes in immune cells, in adenosine concentrations, and this induces the activation of adenosine receptors and ADA [28].

HIV gp120 prevents interaction between CD26, a costimulatory signal for TCR-mediated T cell activation, and ADA; this is implicated in the weak response of T cells to HIV [25]. In addition, when ADA is added to cultures, T cell proliferation is increased.

Our treatment-naïve HIV patients had increased ADA levels, as previously described [29], and treatment with TAF and Triumeq decreased ADA in plasma, though no such decrease was seen with TDF. It has been previously described that serum ADA values are increased in inflammatory conditions such as rheumatoid arthritis, cancer, and tuberculosis [7,8]. In RA, it has been suggested that serum ADA activity acts as a biochemical marker in disease diagnosis and determination of disease activity [10], maintaining and promoting an inflammatory state due to the inhibition of the anti-inflammatory role of adenosine. It is likely that a similar effect is seen in our TDF-treated patients, as the inflammatory state was maintained.

Another possible explanation for this maintenance of increased ADA level in TDF-treated patients is that under antiretroviral therapy, ADA response is affected by a previous depletion of CD4+ T cells [25].

Moreover, in RA, serum ADA reflects monocyte/macrophage activity [9]. Our data indicate a negative association between monocyte counts and ADA values in TDF-treated patients, which also correlates with a decrease in GM-CSF after 12 months of treatment. Previous studies have shown that both monocytes and macrophages [6], but also lymphocytes [4,30], are the main sources of ADA in hematopoietic lineages, with inflammatory diseases playing a crucial role in regulating the release of this enzyme [31]. Previous work has shown that the macrophage function immunomodulator GM-CSF is inhibited by HIV and this produces a modification in the immune dysregulation and cell dysfunction that characterizes HIV-1 infection [32]. Zidovudine (AZT), an NRTI antiretroviral agent analog to thymidine, activity was enhanced by GM-CSF in macrophages, indicating that this factor might be important in elimination of the viral reservoir [33,34]. In addition, since higher ADA activity has been observed in monocytes/macrophages during intracellular infection by HIV due to the release of adenosine [28], we can presume that the ADA levels found in our patients mostly originated from these cells. Taking this into consideration, we can hypothesize that TDF-treated patients have fewer monocytes/macrophages, though these are more active and easily inducible during disease. More data are needed to confirm this observation, and to understand why the same does not happen in TAF-treated patients. This might be attributable to differences in half-life between the two tenofovir compositions in blood. It is believed that this is due to the reduced plasma dose of TAF (25 mg vs. 300 mg of TDF) required to achieve an optimal intracellular concentration of the active metabolite tenofovir diphosphate [35,36]. Furthermore, we cannot underestimate the contributions other cells. It has been observed in DADA 2 (deficiency of adenosine deaminase 2) patients that a compromised B cell compartment may reflect a role played by ADA2 in the bone marrow microenviroment [37].

HIV produces chronic inflammation and immune activation that continues even in the presence of HAART and despite undetectable viral levels and viral latency, indicating that antiretroviral therapy is not able to completely restored the immune function [19,20]. In our cohort, an increase in pro-inflammatory profile after 12 months of treatment was seen in all three groups, increasing the expression of IL2, IL6, IL8, TNFα (in TAF and Triumeq groups) and INFγ (in TDF and TAF groups). Interestingly, however, IL-12 was significantly reduced within the three groups. Previous studies have described HIV-infected patients have increased levels of IL12 receptors in CD4+ T resting lymphocytes, suggesting that IL-12 is a key modulator in HIV-1 infection [38,39]. IL-12 synthesis and release can be selectively inhibited by TNFα during macrophage activation [40]. In reference to the above and in view of our data (Table 2), we observed that when there was a tendency to increase TNFα, there was a trend to a reduction in IL12 concentrations.

Regarding anti-inflammatory cytokines, we found a significant decrease in IL4 for both tenofovir treatments 12 months after treatment initiation when compared to baseline (Table 2), and a non-significant decrease for Triumeq (−51.1 fold, *p* = 0.231). In all treatments, we observed a non-significant decrease in IL10 (Table 2). This aspect has been previously studied [41] and it has been shown that during HIV infection, there is a decrease in the expression of these anti-inflammatory cytokines which is enhanced by antiretroviral therapy and closely related to high levels of IFNγ expression [42,43,44,45]. IFNγ is detected in the acute phase of the infection and is detected throughout the course of infection. In chronic stable disease, IFNγ declines to levels equivalent to healthy controls [43]. Patients in our cohort were in an early stage of the disease, following only 12 months of treatment, which may explain their increased levels of IFNγ. In the case of *Mycobacterium tuberculosis* infection, CD4+ T cells increased with the stimulation of the ADA binding antigen, which correlated with an increase in IFNγ content in these cells [46]. We did not find a significant association between ADA and INFγ, which may be explained by the low number of patients studied.

Interestingly, when we analyzed the correlation between ADA values across different treatments and cytokine levels, an association was found only between ADA and TNFα and IL2 in TDF-treated patients. This may point to the influence of other mechanisms of the virus or HAART in general, which maintain a degree of inflammation that cannot be attributed to changes in ADA or adenosine. During latency, the inflammatory environment not only promotes T-cell proliferation but also stimulates a compensatory response, and a balance between pro- and anti-inflammatory cytokines determines the magnitude and duration of inflammation [20]. During infection, HIV establishes evasion mechanisms by impairing host response, hiding in reservoirs and promoting its own survival and replication [28]. The association found between ADA and TNFα may be a key mediator in the maintenance of inflammation in these patients. It has been described that increased adenosine and activation of the adenosine A2A receptor inhibits both TNFα and IL1β secretion under inflammatory conditions, with an increase in IL10 [47,48], leading to anti-inflammatory effects mediated by adenosine, as occurs in RA [49]. Therefore, the inhibitory effects on the release of TNFα mediated by adenosine through its A2A receptor may be prevented by the increase in ADA and the consequent deamination of adenosine into inosine [28]. A2A receptors are more directly linked to the suppressive/anti-inflammatory effects of adenosine, while A2B also acts as an anchoring molecule to ADA and improves immune responses [28]. Moreover, a positive association between ADA and IL2 could indicate the same, as IL2 promotes TNFα secretion [50].

Although we have not observed a correlation between ADA values and IL13, patients treated with tenofovir (TDF and TAF) had increased expression of this cytokine. Tenofovir, a structural analog of AMP, causes the inhibition of ATP transport through pannexin-1 [51], which causes a decrease in extracellular levels of adenosine. Decreased adenosine concentration in tenofovir-treated patients may lead to an increased release of IL13 as occurs in many other inflammation-derived diseases [52]. It must also be considered, however, that negative feedback loops may occur, as T cell inactivation mediated by increased levels of adenosine may dampen the IL13–initiated inflammatory program.

All these data may explain why increased ADA levels were observed in TDF-treated patients despite the fact that the disease is apparently controlled by antiviral therapy. As mentioned previously, during latency the virus establishes survival mechanisms. Increasing ADA values might be one such mechanism, as an increase in this enzyme is related to a decrease in the immunomodulatory effect of adenosine, and sustained inflammation may be important to maintaining viral reservoirs. Therefore, as we know that tenofovir modulates ATP/adenosine metabolism, the inflammatory stage seen in our patients may be mediated by the antiviral drug by itself, and the increased ADA activity observed in TDF-treated patients compared to the other groups also indicates a regulation of the enzyme mediated by the drug. Further research must be conducted to understand the differences in the impact on ADA among tenofovir compositions, which may be related to the different half-life of TAF and TDF in blood.

The chronic inflammatory state in our patients, together with the increase in ADA values in TDF-treated patients, may provide evidence of how one of the main comorbidities of HIV infection, decreased bone mineral density (BMD), could be regulated by tenofovir in early stages of antiretroviral therapy [53]. Adenosine levels are reduced due to ADA activation, and therefore a proinflammatory state is produced. Adenosine is a key factor in the progression of the disease and changes in CD39 expression slow the progression to AIDS, implicating adenosine production in disease progression [26]. Adenosine A1, A2A, and A2B receptors anchor to ADA [54,55]. The A1 receptor requires the lowest adenosine concentration and is constitutively active in bone and induces osteoclastogenesis and bone resorption [56,57]. Tenofovir may exert this bone degradation effect by activating the A1 receptor, as adenosine levels are decreased due to both pannexin-1 blockade [51] and ADA activation (as shown in this manuscript), with a detrimental effect on A2A receptor activation, the receptor involved in inhibition of bone loss [58].

Finally, the possible role of adenosine deaminase acting on RNA (ADAR) must be taken into consideration, as these enzymes produce adenosine-to-inosine deamination in regions of the RNA, and play important roles during viral infections [59]. ADAR can have both a pro- and anti-viral effect depending on virus-host combination and how editing is done in the RNA. These enzymes also trigger the innate immune response [60]. In the case of HIV it has been shown that ADAR1, an isoenzyme regulated by IFN, plays a pro-viral role, enhancing HIV-1 expression and replication [59]. As during HIV replication, the virus uses the host cell machinery, and the activation of ADAR might be important to this process. A novel aspect to explore in the future is how tenofovir acts on these enzymes, as once tenofovir is transported into the cells and once it is polyphosphorylated, it binds to the HIV-1 reverse transcriptase by competing with the natural substrate. It would be beneficial to understand whether ADAR is altered in this situation and if this may alter the inflammatory stage of HIV patients.

In conclusion, ADA values in HIV patients can be used as a biomarker of the progression of the disease. Levels of ADA may be modulated by the antiretroviral therapy of choice, affecting the associated comorbidities (fibrosis, bone injury). Tenofovir (concretely TDF) may potentiate both the inflammatory profile and the consequent degradation of adenosine by ADA and its associated deleterious effects.

## 4. Materials and Methods

### 4.1. Subject and Design—Inclusion and Exclusion Criteria and Ethical Aspects

We included 92 adult men recently diagnosed with HIV infection who were naïve to treatment at the initiation of the study. Patients were enrolled between May 2016 and September 2017 in the Infectious Disease Division in Hospital Fundación Jimenez Díaz, a teaching hospital in Madrid, Spain. Patients were grouped according to the antiviral treatment they received, the decision for which was based on medical criteria, i.e., (1) TDF (Gilead Sciences, Foster City, CA, USA) 245 mg/day (*n* = 21), (2) TAF (Gilead Sciences, Foster City, CA, USA) 10 mg/day (*n* = 22), and (3) abacavir/dolutegravir/lamivudine in combination (Triumeq) (ViiV Heathcare, Brentford, UK) 600 mg/day (*n* = 39). The protocol for this study was approved by the clinical research ethics committee (CEIm) of the Hospital Fundación Jimenez Diaz (approval code: PIC 155-2016, approved on 20 December 2016) and is in adherence with the tenets of the Declaration of Helsinki. All patients provided signed informed consent before being included in the study.

Patients not fulfilling the following criteria were excluded: age over 50 years, previous antiretroviral treatment, bone treatment (denosumab, vitamin D), diabetes, corticosteroid treatment, rheumatic diseases, renal failure, thyrotoxicosis, advanced liver disease, malabsorption syndrome, or neoplasias.

A second limitation concerns the lack of a study group consisting of individuals not infected with the HIV virus; nonetheless, our results can be contrasted with well-established findings from studies conducted in the general population. Though our cohort consists of individuals who have lived with HIV infection for a short time, certain bias may have been introduced in this regard, as we cannot rule out a slight impact of HIV infection on metabolism in the first stages of infection. However, the fact that over 90% of patients had stage-A disease indicates an appropriate degree of homogeneity.

### 4.2. Measurements and Reference Values

Blood was collected at baseline and then at 3 and 12 months after the initiation of treatment under fasting conditions. ADA determination was performed using a kinetic colorimetric assay (Adenosine Deaminase Assay kit # DZ117A-K, DIAZYME Laboratories, Dresden, Germany) based on the enzymatic deamination of adenosine to inosine in the Department of Clinical Analysis and Biochemistry at Hospital Fundación Jimenez Díaz. For humans, normal ADA reference values for serum and plasma range from 0 to 15 U/L. Counts of monocytes and neutrophils were obtained from the corresponding clinical records derived from studies of blood samples.

One limitation of the study is the number of patient samples collected at each time point. For some procedures we were unable to obtain samples from all patients.

### 4.3. Cytokines Measurement by MILLIPLEX^®^ Multiplex Assays Using Luminex^®^

We used the MILLIPLEX MAP Human High Sensitivity T Cell Magnetic Bead multiplex kit (Merk Millipore, Billerica, MA, USA) to measure plasma concentrations of pro-inflammatory (IL1β, TNFα, IL8, IL12 (p70), INFγ (interferon gamma), GM-CSF (granulocyte-macrophage colony-stimulating factor), IL6, IL7, IL2) and anti-inflammatory cytokines (IL10, IL13, IL4, IL5). Samples were analyzed in duplicate following manufacturer recommendations. Briefly, after centrifugation (15 min at 2500× g), plasma aliquots were kept at -80°C until used. ELISA multiplex was done following manufacturer protocol, including beads preparation, standard and quality controls. Each sample was analyzed in duplicate. by LUMINEX MAGPIX^®^ technology (Luminex Corp., Austin, TX, USA). The limits of detection were as follows: GM-CSF, 0.6 pg/mL; INFγ, 0.94 pg/mL; IL10, 0.93 pg/mL; IL12 (p70), 0.27 pg/mL; IL13, 0.34 pg/mL; IL1β, 0.24 pg/mL; IL2, 0.30 pg/mL; IL4, 1.84 pg/mL; IL5, 0.22 pg/mL; IL6, 0.17 pg/mL; IL7, 0.6 pg/mL; IL8, 0.25 pg/mL; and TNFα, 0.21 pg/mL. Intra-assay coefficients of variation for the cytokines studied were less than 5%; inter-assay coefficients of variation were less than 15% for both multiplex kits.

### 4.4. Statistical Analysis

Qualitative variables were expressed as frequencies and percentages, whereas quantitative variables appear as mean and standard deviation, or median and interquartile range, according to the results of a normality test (Kolmogorov–Smirnov test). Comparisons between quantitative variables were performed using Student’s *t* test, Chi square test, or Fisher’s exact test. Cytokines and changes in ADA are described as median values and quartiles (percentiles of 25% and 75%) and are compared using the Wilcoxon rank test. Correlation between variables was studied using Spearman’s rank correlation coefficient. For all determinations, we used R software version 3.6.0 (R Core team (2020); R Foundation for Statistical Computing, Vienna, Austria), and statistical significance was set at *p* < 0.05.

## Figures and Tables

**Table 1:** ADA (U/L) plasma concentrations at baseline, 3- and 12-months post-treatment. It represented median (minimum value, maximum value). * *p* < 0.05 vs baseline.

ADA (U/L) Plasma Concentrations			
Treatment	Baseline	3 Months	12 Months
TDF	15.43 (12.76, 17.94) (*n* = 20)	11.72 (9.55, 14.95) (*n* = 17)	13.84 (12.53, 16.69) (*n* = 13)
TAF	17.66 (13.50, 22.31) (*n* = 13)	14.87 (12.52, 17.81) (*n* = 16)	9.30 (7.95, 12.72) (*n* = 4) *
Triumeq	15.46 (13.81, 21.60) (*n* = 37)	13.36 (11.55, 17.92) (*n* = 33)	12.68 (11.59, 14.75) (*n* = 23)

**Table 2:** Cytokine increments at 12 months after treatment compare to baseline. Median values (interquartil range) and significance (*p*) vs. baseline are shown. *p1*: TDF vs. TAF, *p2*: TDF vs. Triumeq, *p3*: TAF vs. Triumeq. Wilcoxon rank sum test. *p* < 0.05 was considered significant.

	Cytokine Increments Baseline: 12 Months Post-Treatment								
	TDF		TAF		Triumeq				
Variable	Median (*Q1*, *Q3*)	*p*	Median (*Q1*, *Q3*)	*p*	Median (*Q1*, *Q3*)	*p*	*p1*	*p2*	*p3*
**Pro-Inflammatory Cytokines**									
*TNFα*	−5.6 (−23.1, 33.6)	0.893	44.8 (3.2, 81.3)	0.007	1.8 (−29.0, 42.3)	0.609	0.075	0.907	0.091
*IFNγ*	19.2 (−39.2, 69.6)	0.358	30.5 (−10.4, 62.4)	0.055	−11.1 (−44.6, 55.9)	0.228	0.627	0.663	0.224
*IL1β*	−1.9 (−39.2, 17.1)	0.946	28.9 (−32.9, 64.0)	0.170	−12.4 (−56.4, 30.7)	0.902	0.395	0.711	0.235
*IL2*	33.6 (−6.2, 113.4)	0.042	62.1 (6.9, 106.6)	0.002	21.7 (−17.5, 77.8)	0.336	0.553	0.782	0.267
*IL6*	55.3 (−2.9, 213.6)	0.03	129.9 (4.0, 222.1)	0.005	2.4 (−49.3, 119.6)	0.540	0.837	0.218	0.083
*IL8*	41.0 (−1.5, 152.5)	0.078	108.5 (45.4, 207.3)	0.000	27.9 (−31.3, 129.1)	0.337	0.179	0.497	0.015
*IL12*	−4.0 (−44.8, 33.7)	0.305	−15.1 (−43.4, 34.4)	0.651	−36.0 (−59.0, 3.5)	0.086	0.734	0.293	0.267
**Anti-Inflammatory Cytokines**									
*IL4*	−35.7 (−62.9, −3.4)	0.005	−59.4 (−74.2, −46.9)	0.008	−51.1 (−65.3, -18.0)	0.231	0.255	0.636	0.315
*IL10*	−20.3 (−56.2, 15.0)	0.153	−7.0 (−39.4, 90.3)	0.468	−44.1 (−65.0, 2.3)	0.378	0.180	0.468	0.036
*IL13*	13.6 (−61.6, 81.3)	0.542	32.6 (−35.9, 112.2)	0.120	−43.5 (−81.4, 34.0)	0.144	0.316	0.427	0.035
**Colony Stimulating Factors**									
*IL5*	−17.2 (−53.3, 35.6)	0.855	78.4 (−31.5, 160.7)	0.027	−25.0 (−53.6, 54.3)	0.309	0.084	0.983	0.055
*IL7*	56.2 (−7.0, 192.9)	0.035	−26.4 (−41.0, 12.7)	0.156	−2.9 (−35.9, 58.0)	0.586	0.010	0.503	0.294
*GM-CSF*	−35.1 (−57.7, −25.4)	0.001	7.5 (−28.3, 42.7)	0.490	−37.8 (−60.1, 18.4)	0.935	0.009	0.325	0.147

**Table 3:** Variation in monocytes and neutrophils between baseline and 12 months post-treatment. Mean cell/µL (SEM) is represented. * *p* < 0.05 vs baseline.

**Monocytes/µL on Blood Samples**		
**Treatment**	**Baseline**	**12 Months**
TDF	414 (0.02951) (*n* = 21)	401 (0.02712) (*n* = 21)
TAF	404 (0.02712) (*n* = 22)	450 (0.02255) (*n* = 22) *
Triumeq	378 (0.01604) (*n* = 39)	403 (0.02104) (*n* = 39)
**Neutrophils/µL on Blood Samples**		
**Treatment**	**Baseline**	**12 Months**
TDF	3205 (0.20620) (*n* = 21)	3595 (0.31350) (*n* = 21)
TAF	3223 (0.2136) (*n* = 22)	3641 (0.3270) (*n* = 22)
Triumeq	3495 (0.2538) (*n* = 39)	3873 (0.2599) (*n* = 39)

## Supplementary Materials

Supplementary materials can be found at https://www.mdpi.com/1422-0067/21/10/3590/s1.
