# Supplementary material for: Adenosine Deaminase as a Biomarker of Tenofovir Mediated Inflammation in Naïve HIV Patients
Source: Int J Mol Sci. 2020 May 19;21(10):3590. doi: 10.3390/ijms21103590 (PMC7278965; doi:10.3390/ijms21103590)
Supplement: Supplementary file 1 [file ijms-21-03590-s001.pdf]

Suplemmental Table 1. Evolution of the ADA concentrations by treatments in form of increases. It represents median and interquartile range. – indicates decrease. p1: comparing with baseline, p2: comparing between treatment. Wilcoxon rank sum test.

	**TDF**		**TAF**		
**Evolution**	**Median (Q1, Q3)**	**p^1**	**Median (Q1, Q3)**	**p^1**	**p^2**
**3 months**	-20 (-29.4, -12.5)	0.000	-3.8 (-18.2, 26.9)	0.860	0.015
**12 months**	-7.0 (-19.8, 6.2)	0.455	-49.7 (-54.6, -29.0)	0.000	0.001
	**TDF**		**Abacavir**		
**3 months**	-20 (-29.4, -12.5)	0.000	-20.8 (-29.4, 0.0)	0.078	0.421
**12 months**	-7.0 (-19.8, 6.2)	0.455	-30.5 (-48.9, -14.3)	0.000	0.006
	**TAF**		**Abacavir**		
**3 months**	-3.8 (-18.2, 26.9)	0.860	-20.8 (-29.4, 0.0)	0.078	0.134
**12 months**	-49.7 (-54.6, -29.0)	0.000	-30.5 (-48.9, -14.3)	0.000	0.101
